# Quantum physical analysis of caffeine and nicotine in CCL4 and DMSO solvent using density functional theory

**DOI:** 10.1038/s41598-025-91211-9

**Published:** 2025-03-26

**Authors:** Manoj Sah, Raju Chaudhary, Suresh Kumar Sahani, Kameshwar Sahani, Binay Kumar Pandey, Digvijay Pandey, Mesfin Esayas Lelisho

**Affiliations:** 1 Department of Physics, St. Xavier’s College, Maitighar, Kathmandu, Nepal; 2 Department of Science and Technology, Rajarshi Janak University, Janakpurdham, Nepal; 3 Department of Civil Engineering, Kathmandu University, Kathmandu, Nepal; 4 Department of Information Technology, College of Technology, Govind Ballabh Pant University of Agriculture and Technology Pantnagar, Udham Singh Nagar, Uttrakhand India; 5 Department of Technical Education (Government of U.P), Lucknow, India; 6 Department of Statistics, College of Natural and Computational Science, Mizan-Tepi University, Tepi, Ethiopia

**Keywords:** Raman spectroscopy, UV-Vis, NLO, AIM, RDG, ELF, LOL, Neurochemistry, Gastroenterology, Signs and symptoms, Chemistry, Physics

## Abstract

This work used the 6-311++G(d, p) basis set in the DFT/B3LYP and DFT/CAM-B3LYP technique to build the molecular structures of the nicotine and caffeine molecules. The minimum energy gives stability to these molecules with their corresponding dipole moment. The optimized structure to compute Raman spectroscopy and UV-Vis in CCl4 and DMSO solvent, employing the basis set 6-311++G(d, p), the DFT/B3LYP and CAM-B3LYP hybrid function, with the C-PCM model. The re-optimized molecule is used to study NLOs property which also give the dipole moment, polarizability and hyperpolarizability of titled molecules. We used AIM to investigate these molecules’ intramolecular interactions, bond critical points, and interbasin paths. Multiwfn software 3.8 produces the NCI-RGD diagram, which we use to determine weak interaction, electron density, Van der Waals interaction, steric effect, and hydrogen bond. Similarly, we analyze the covalent bond with the molecular surface using ELF and LOL techniques.

## Introduction

Caffeine and nicotine are considered alkaloids and therapeutic drugs. They stimulate the human central nervous system^1^. Coffee, tea, cocoa beans, and yerba mate naturally contain caffeine. However, cigarettes, cigars, snuff, and pipe tobacco are the main sources of nicotine^2^. Globally, people consume caffeine, an organic compound, due to its stimulating properties in the brain that increase alertness and decrease tiredness^3^. They have beneficial effects on humans and, when taken in moderation, can reduce headaches, depression, and constipation. However, excessive use of caffeine can lead to fatal consequences, similar to other drugs^4^. An excessive quantity of caffeine in the body triggers a toxic condition that manifests as insomnia, heart palpitations, vomiting, and rapid breathing^5^. Nicotine raises the risk of stroke and can cause an increase in heart rate, blood pressure, and heart blood flow^6^. The most popular drinks in Asian countries are coffee and tea, considered major sources of caffeine and exhibiting dose-dependent effects^7^. The high concentrations of caffeine can cause faintness, temporary cognitive impairment, migraines, and even death due to its low boiling point and dissolve easily in water^8^. Most drugs consist of small quantities of caffeine, and normal caffeine consumption enhances performance on tasks that require alertness, such as simulated driving. It also reduces fatigue and may elevate the mood during mental activities^9^.

The chemical formula for caffeine (1, 3, 7-trimethyl-3, 7-dihydro-1 H-purine-2, 6-dione) acts as a natural stimulant highly soluble in water. Similarly, nicotine (3-(1-methyl pyrrolidine-2-yl) pyridine) is a moderately water-soluble organic compound with the molecular formula C10H14N2^10^. Since nicotine is a cardioactive drug, regular use of cigarettes and chewing tobacco increases nicotine levels, which can alter heart rate and blood pressure^11^. Nicotine’s addictive nature paralyzes the neurological system^12^. It may cause cancer because of its effects on DNA, the immune system, and cells^13^. Several computational studies have investigated the two stimulant molecules, caffeine and nicotine. A. Belay et al. used a UV-Vis spectrophotometer to measure the transitional dipole moment and molar decadic absorption coefficients of pure caffeine in water and dichloromethane^14^. C. Cappelli et al. studied Raman and UV-Vis spectra of caffeine in water solvent and non-covalent intermolecular interaction; in contrast Peter M. Clayton et al. studied nicotine UV absorption spectra in the range between 300 nm and 200 nm with water solvent/aqueous solution^15,16^. Similarly, R. Rijal et al. have investigated vibrational spectroscopy of both caffeine and nicotine in the gas phase and water solvent^17^. Computational chemistry is vital in investigating the relationship between chemistry and biophysics. Many scientists and researchers have focused a lot of attention on computational chemistry to tackle real-world issues and comprehend how drugs affect protein structure and transport mechanisms. These research projects motivated us to explore the caffeine and nicotine molecules. We used the Density Functional Theory (DFT) method with the B3LYP/6-311++G(d, p) and CAM-B3LYP/6-311++G(d, p) basis sets to generate the optimized molecules and studied their physical characteristics such as AIM, NCI-RDG analysis, ELE, LOL, and electron density. Similarly, non-linear optical properties and vibrational spectroscopy, such as Raman and UV-Vis spectroscopy, were studied and compared for the same parameters. To the best of our knowledge, the molecules of caffeine and nicotine have not yet been thoroughly investigated computationally in CCl4 and DMSO solvents.

## Materials and methods

All the essential computational physical calculations were performed using the Gaussian09W software package utilizing the 6-311++G(d, p) basis set^18^. These computational calculations were carried out using density functional theory (DFT) and the Becke-Lee-Yang-Parr (B3LYP) and Coulomb Attenuated Method (CAM)-B3LYP functions^19,20^. The Gauss View 6.0.16 software package^21^ utilized computational methods to generate the stabilized molecular structure of these title molecules. Further, the geometrically optimized structures were used to perform vibrational and topological calculations. The minimum total energies of the title molecules were re-optimized for Raman and UV-Vis spectra in the gas phase in CCl4 and DMSO solvents by using the conductor-like polarizable continuum model (C-PCM) to find molecular free energies and their corresponding properties in different phases^22^. The Raman activities (*S*_*i*_) were obtained using the Gaussian09 program with the following relationship of Raman scattering:

1 $$I_{i}=\frac{[f{(\nu 0-\nu i)}^{4}{S}_{i}]}{\left[\nu i\right\{1-\text{e}\text{x}\text{p}(-\frac{hc\nu i}{kT})\left\}\right]}$$

Where $\nu 0$ represents the wave number in cm^−1^ of the exciting light, $\nu i$ represents the vibrational wave number of the ith normal mode; also, h, c and k are Plank’s constant, the speed of light, and the Boltzmann constant, respectively. The dipole moment, mean polarizability (${\alpha}_{\text{t}\text{o}\text{t}})$ and the first static hyperpolarizability (β_tot_) of the given molecules are calculated in terms of x, y, and z components in the following equations:

2 $${\mu}_{tot}={({\mu}_{x}^{2}+{\mu}_{y}^{2}+{\mu}_{z}^{2})}^{1/2}$$

3 $${\alpha}_{tot}=1/3[{\alpha}_{xx}+{\alpha}_{yy}+{\alpha}_{zz}]$$

4 $$\begin{aligned}{\beta}_{tot}&={\left[{\beta}_{x}^{2}+{\beta}_{y}^{2}+{\beta}_{z}^{2}\right]}^{1/2} \\ &={\left[{\left({\beta}_{xxx}+{\beta}_{xyy}+{\beta}_{xzz}\right)}^{2}+{\left({\beta}_{yyy}+{\beta}_{yzz}+{\beta}_{yxx}\right)}^{2}+{({\beta}_{zzz}+{\beta}_{zxx}+{\beta}_{zyy})}^{2}\right]}^{1/2}\end{aligned}$$

where, µ_x_^2^, µ_y_^2^ and µ_z_^2^ are components of dipole moment. The polarizability and hyper-polarizability tensors (α_xx_, α_xy_, α_yy_, α_xz_, α_yz_, α_zz_ and β_xxx_, β_xxy_, β_xyy_, β_yyy_, β_xxz_, β_xyz_, β_yyz_, β_xzz_, β_yzz_, β_zzz_) respectively. The obtained output values of α and β are in atomic units (a.u). These units are converted into electrostatic units (esu), for (α; 1 a.u = 0.1482 × 10^−24^ esu, β; 1 a.u = 8.6393 × 10^−33^ esu). Also, the TD-DFT approach with the same basis set was employed to represent the UV-Vis absorption spectra with maximum wavelength. Different topological properties of these title molecules were studied by using the Multiwfn 3.8 software package. The AIM (atom in a molecule), NCI-RDG, and ELF & LOL of caffeine and nicotine molecules are obtained using Multiwfn software 3.8^23^.

## Results and finding

### Optimized geometrical structure

**Fig. 1:**
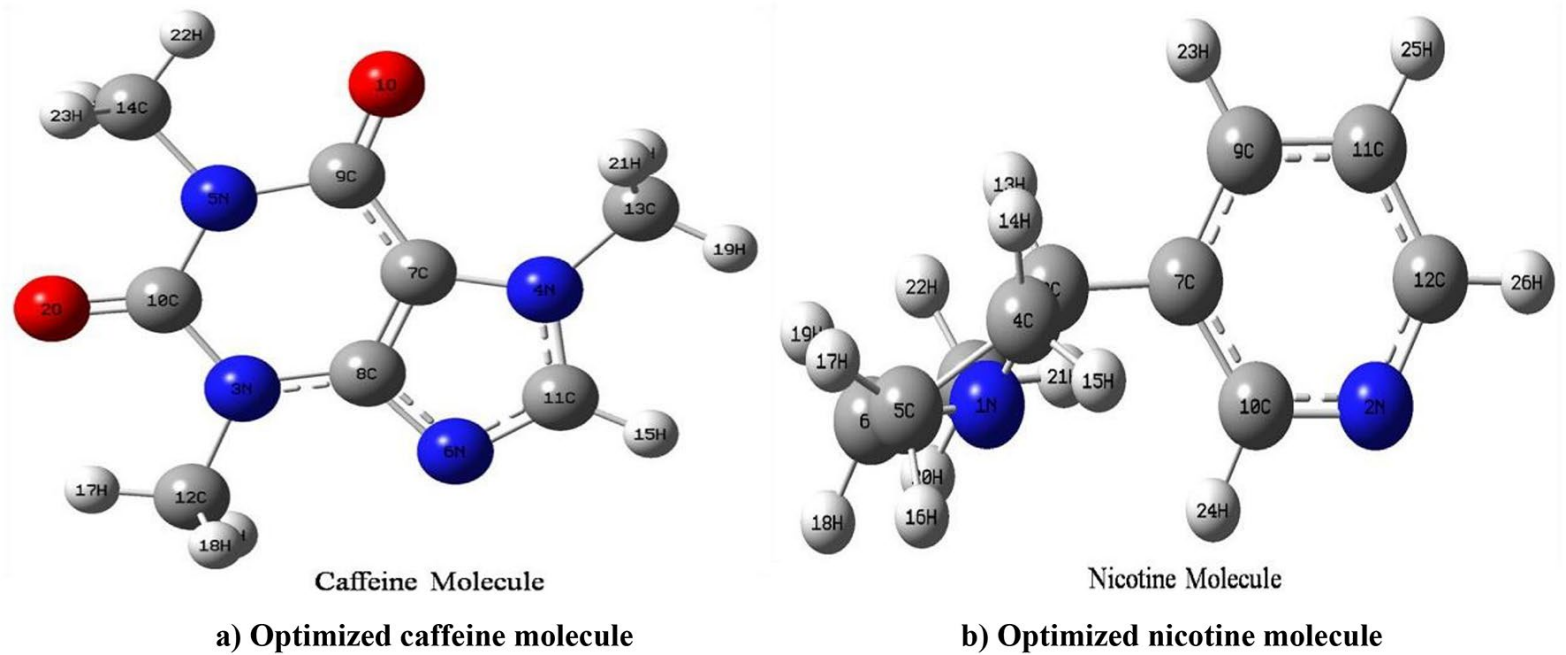
Geometrically optimized structure of (**a**) caffeine and (**b**) nicotine molecule with atomic symbol and labeling of atoms.

The Gaussian 09 W software is designed to optimize the molecules of caffeine and nicotine. The calculated minimum total energy to stabilize the molecule in various phases, such as the gas phase, CCl4 (carbon tetrachloride), and DMSO (dimethyl sulfoxide) solvent. The comparison of two stimulant molecules, caffeine and nicotine, in different solvents like CCl4 and DMSO shows polar and non-polar nature. The comparison of the dipole moment between two molecules in different solvents reveals that a higher value indicates a polar nature. The two molecules, nicotine and caffeine, are compared and studied in two different solvents, CCl4 and DMSO. The comparison of dipole moment and minimum energy of caffeine and nicotine molecules was compared in different phases. The lowest total energies of caffeine and nicotine molecules found in the gas phase are − 18518.8 eV and − 13581.43 eV, respectively. Also, the comparison of minimum energy between B3LYP and CAM-B3LYP shows higher stability of molecules in different phases. Similarly, the lowest energy for caffeine is smaller than nicotine in CCl4 and DMSO solvents. The caffeine molecule is more stable in the gas phase, and the stability of the molecule is continuously decreasing from neutral gas phase e to DMSO solvent. Similarly, the stability of the nicotine molecule decreases from the neutral gas phase to the DMSO solvent Fig. 1 displays the optimized geometries of the caffeine and nicotine molecules, along with their associated symbols and atom labels. Table 1 lists all the minimum energies of nicotine and caffeine molecules with their corresponding dipole moments in gas phases, CCl4, and DMSO solvents.

According to^24^ increasing the dielectric constant in caffeine molecules can make the dipole moment rise faster from a neutral gas phase to the DMSO solvent. In the same way, the nicotine molecule’s dipole moment is 2.97 Debye in the neutral gas phase. It is higher in the CCl4 solution than in the gas phase, and it is much higher in the DMSO solvent than in either the gas phase or the CCl4 solution. The comparison of dipole moments between B3LYP and CAM-B3LYP gives higher dipole moments in CAM-B3LYP hybrid functions except in caffeine in CCl4 solvent.

**Table 1:** The minimum energy and their corresponding dipole moment of nicotine and caffeine molecules in the gaseous phase, in CCl4 and DMSO solvent.

Different states	B3LYP		CAM-B3LYP	
	Minimum energy (eV)	Dipole (Debye)	Minimum energy (eV)	Dipole (Debye)
Caffeine in gas phase	− 18518.8	3.98	− 18510.69	4.04
Caffeine in DMSO	− 18519.4	5.44	− 18511.09	5.48
Caffeine in CCL4	− 18519.2	4.86	− 18510.90	4.74
Nicotine in gas phase	− 13581.43	2.97	− 13574.01	3.05
Nicotine in DMSO	− 13581.80	4.33	− 13574.20	4.41
Nicotine in CCL4	− 13581.69	3.60	− 13574.11	3.69

### Vibrational spectroscopic analysis

Vibrational spectroscopy provides a dynamic image^25,26^ of the molecule based on the vibrational energy level of the sample. The infrared and Raman spectroscopy methods are the most essential approaches used in vibration spectroscopy^27,28^. Caffeine and nicotine molecules consist of 24 and 26 atoms, respectively. Using the 6-311G++(d, p) basis set, vibrational spectroscopy was used to figure out the Raman and UV-Vis of caffeine and nicotine molecules in CCl4 and DMSO solvents. After full optimization with the real minimum potential energy, different vibrational modes were created. The Raman and UV-Vis spectra revealed no imaginary vibrational modes^29,30^. The Raman spectroscopy calculated various vibration modes when the solvents CCl4 and DMSO were present. The range of wavenumber ranges between 0 and 3500 cm^−1^ in both CCl4 and DMSO solvents.

#### Raman spectra analysis

Nonpolar molecules use Raman spectroscopy for symmetric vibration. Raman spectroscopy is used to look into vibrational, rotational, and other low-frequency modes in molecular bonding in materials that are very sensitive to changes in their structure^31,32^. In Raman analysis, the fingerprint region lies between 400 and 1800 cm^−1^. The highest peak in Raman activity shows the higher polarizability of the molecule in a nonpolar bond. The strong Raman activity has been seen at 3000–3150 cm^−1^ in the caffeine molecule in CCl4 and DMSO solvents. Also, strong Raman activity has been observed at 2900–3200 cm^−1^ in the nicotine molecule.

**Fig. 2:**
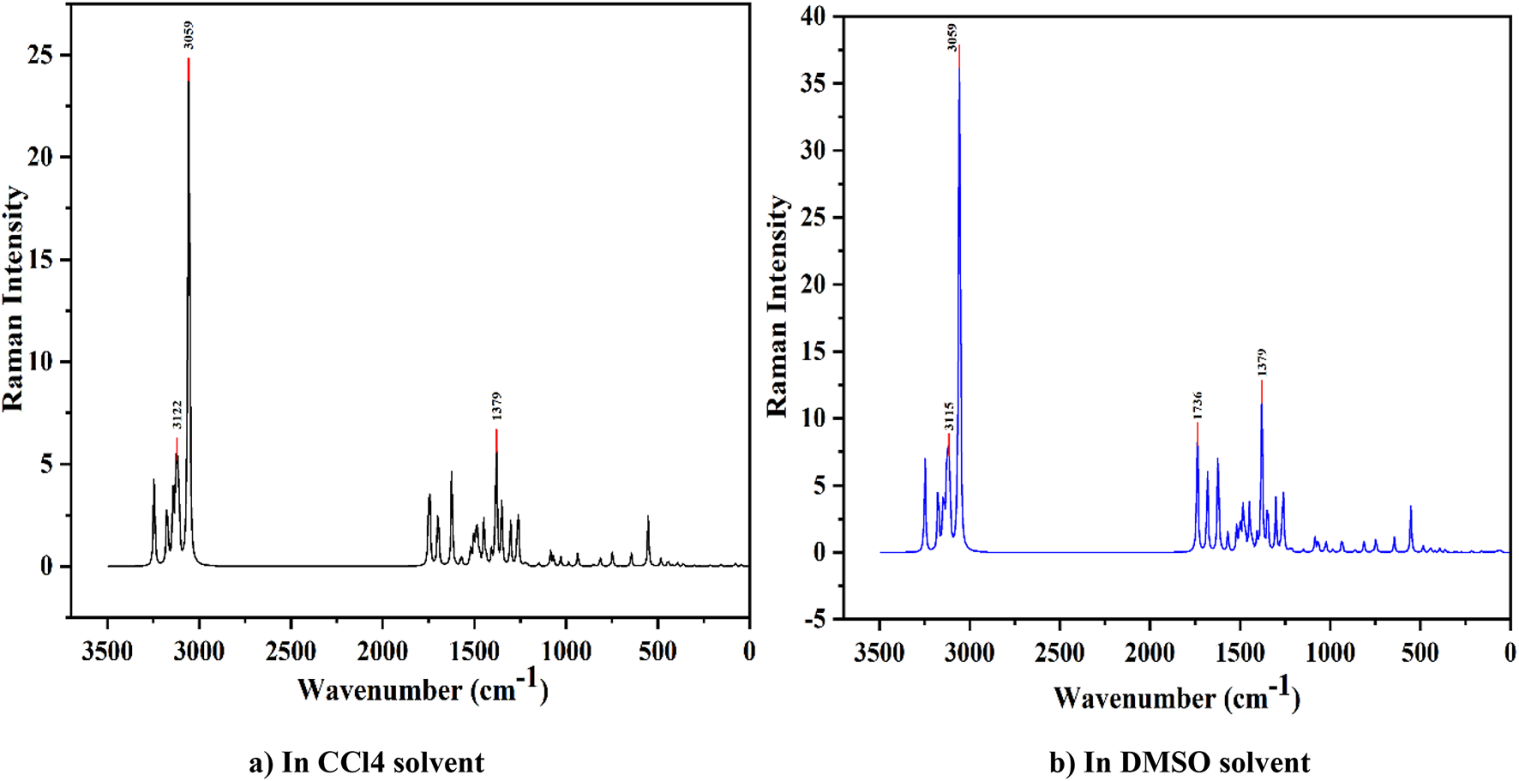
Raman spectra of the caffeine molecule in (a) CCL4 and (b) DMSO solvent.

**Fig. 3:**
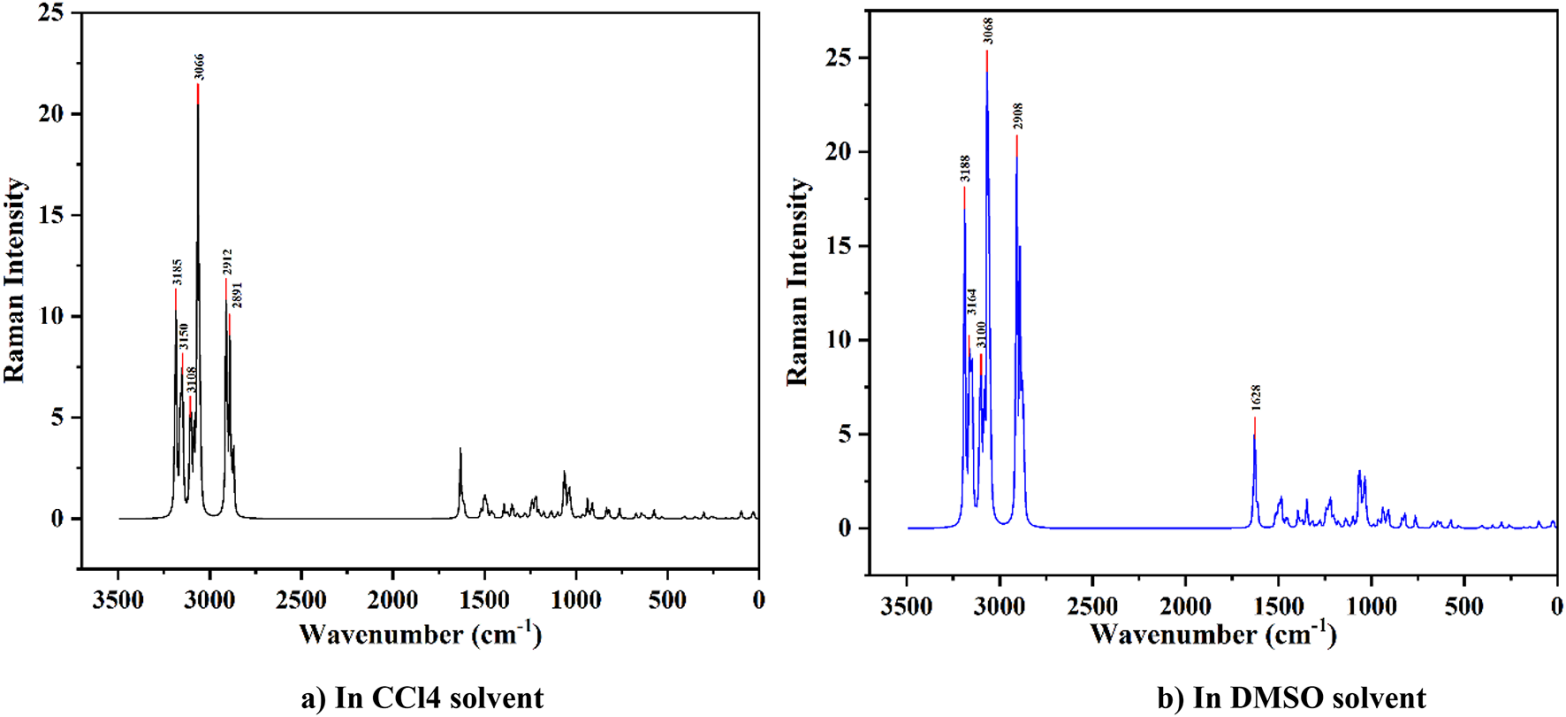
Raman spectra of the nicotine molecule in (**a**) CCL4 and (**b**) DMSO solvent.

In this study, Fig. 2, the Raman activity of the caffeine molecule has a cluster of peaks around 600–1750 cm^−1^, which is the fingerprint region, and the highest peaks were found around 3100–3250 cm^−1^ for both caffeine and nicotine molecules in CCl4 and DMSO solvents. The highest peak is at 3059 cm^−1^ in both CCl4 and DMSO solvents. The highest peak is at 3059 cm^−1^ in both CCl4 and DMSO solvents. This is caused by C–H stretching modes of vibration. The caffeine molecule has two other peaks at 3122 cm^−1^ and 3115 cm^−1^, which are also caused by C–H stretching modes of vibration. Similarly, in Fig. 3, the fingerprint region of the nicotine molecule’s Raman activity lies between 900 and 1650 cm^−1^, while both CCl4 and DMSO solvents exhibit the highest peak around 2900–3200 cm^−1^. There is a lot of Raman activity at 3066 cm^−1^, which is caused by C-H stretching modes in the CCl4 solvent. There is also a peak around 3068 cm^−1^ in the nicotine molecule where C–H modes of vibrations cause vibrations^33,34^. Both caffeine and nicotine molecules primarily exhibit C–H modes of vibration. The structure of the caffeine molecule is smaller than the nicotine molecule, so it has fewer high peaks than the nicotine molecule. The nicotine molecule has a more complicated structure than the caffeine molecule.

#### Ultraviolet-visible (UV-Vis) absorption spectra

**Fig. 4:**
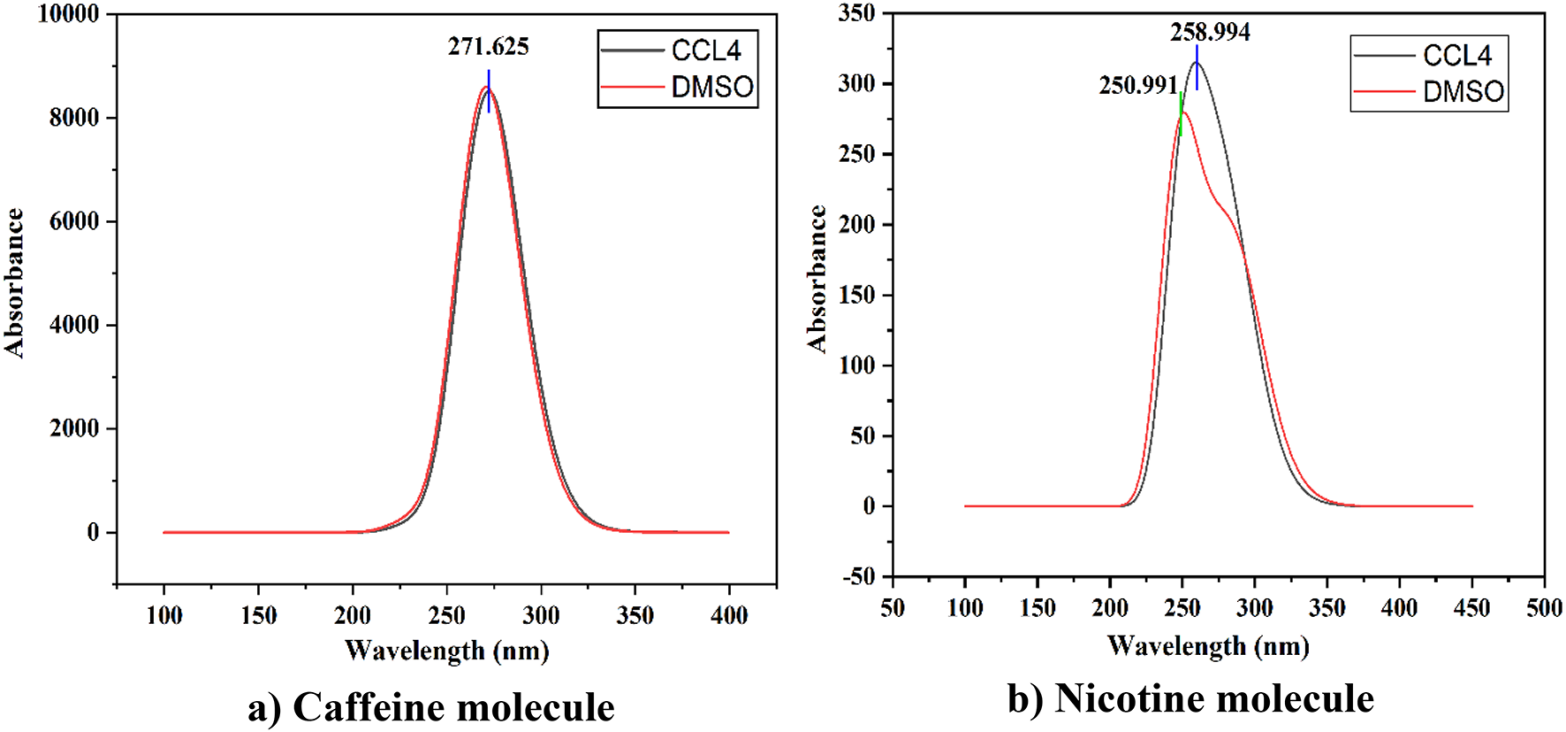
UV-Vis spectra of (**a**) caffeine with CCL4 and DMSO solvent and (**b**) nicotine with CCL4 and DMSO solvent.

UV-Vis spectral analysis has investigated the nature of the electrical transition properties in the caffeine and nicotine molecules^35^. Using the TD-DFT method, we obtained the absorbance spectra of nicotine and caffeine in the CCl4 and DMSO solvents at the 6-311++G(d, p) basis set, as shown in Fig. 4. To analyze the effects of solvents, the C-PCM model was presented^36^. The maximum wavelength shows the absorbance of light from caffeine and nicotine molecules. In this work, the graphical maximum wavelength (λmax) for caffeine molecules in both CCl4 and DMSO solvents to be 271.625 nm. Also, the maximum wavelength for the nicotine molecule in CCl4 solvent is 258.994 nm, and 250.991 nm for DMSO solvent; these fall into the middle UV region of the electromagnetic spectrum. There is a minor deviation from the estimated maximum peak absorbance for the nicotine molecule in CCl4 and DMSO solvent. The absorbance of the caffeine molecule in CCl4 and DMSO solvent is quite similar, but for the nicotine molecule, the absorbance is slightly different in CCl4 and DMSO solvent, which is about 8.003 nm. The nicotine molecule absorbs more light in the CCl4 solvent phase than DMSO.

The highest peak absorbance of the caffeine and nicotine molecules is shown in Fig. 4 with CCl4 and DMSO solvent, which resembles the experimental findings published by Belay et al.^14^. Table 2 tabulates the calculated values of electronic transition values. The calculated maximum wavelength for the first excited state of the caffeine molecule shows the highest peak at 272.39 nm and 270.90 nm in CCl4 and DMSO solvent. Similarly, for the nicotine molecule, the first excited state shows the highest peak around 284.04 nm and 288.69 nm in CCl4 and DMSO solvent, respectively. The comparison of the graphical value with the calculated value is quite different in the first excited state of the nicotine molecule but similar for the caffeine molecule. The third excited state of the nicotine molecule has wavelengths of 252.49 nm and 248.23 nm, which resemble with UV-Vis graphical values of 258.99 nm and 250.99 nm.

**Table 2:** The electronic transition of the first excited state occurs when caffeine and nicotine are assigned to different phases.

Different phases	States S0→S1	Assignment	Coefficient	Energy (eV)	Wavelength (nm)	Oscillator strength	Calculated value (nm)
Caffeine in CCL4	51 → 52	HOMO → LUMO	0.69316 (80.17%)	4.5518	272.39	0.2100	271.62
Caffeine in DMSO	51 → 52	HOMO → LUMO	0.69246 (95.90%)	4.5768	270.90	0.2121	271.62
Nicotine in CCL4	42 → 45	HOMO-2 → LUMO	− 0.12613 (3.18%)	4.3651	284.04	0.0040	258.99
	44 → 45	HOMO → LUMO	0.69451 (96.46%)				
Nicotine in DMSO	44 → 45	HOMO → LUMO	0.70090 (98.25%)	4.2947	288.69	0.0037	250.99

### Non-linear optical properties (NLO)

The dipole moment, polarizability, and hyperpolarizabilities of organic molecules are significant response qualities. These compounds, possessing hyperpolarizabilities, declare themselves as components of non-linear optical materials. The hyperpolarizability of NLOs is coherent optical mechanisms that change shape nonlinearly with the brightness of the light that hits them^37^. This makes them very useful in large optical fields. Non-linear optical properties have enormous significance in the field of biomedical imaging as well as pharmacology^38^.

**Table 3:** The non-linear optical (NLO) properties of nicotine and caffeine molecules in different phases with electrical dipole moment (µ_tot_), polarizability (α_tot_) and hyperpolarizability (β_tot_).

Parameters	Nicotine			Caffeine		
	CAM-B3LYP/6-311++G(d, *p*)			CAM-B3LYP/6-311G++(d, *p*)		
	Gas phase	CCl4	DMSO	Gas phase	CCl4	DMSO
Dipole moment	Debye			Debye		
µ_x_	− 1.5070	− 1.7220	− 1.9313	− 3.9859	− 4.6629	− 5.3778
µ_y_	− 0.6822	− 0.6398	− 0.1811	0.7159	0.8865	1.0871
µ_z_	2.5648	3.2015	3.9668	− 0.0005	− 0.0007	− 0.0010
µ_tot_	3.0520	3.6911	4.4156	4.0497	4.7464	5.4866
Polarsability (α_0_)	a.u			a.u		
α_xx_	159.0148	180.2401	201.2722	153.4388	176.0002	199.0851
α_xy_	0.3989	0.90615	1.9486	− 0.7970	− 1.20721	− 1.8397
α_yy_	102.8924	118.416	134.0620	143.8400	169.4734	197.6394
α_xz_	0.9495	0.7373	0.3030	0.0055	0.0066	0.0077
α_yz_	− 4.6672	− 4.6348	0.3939	0.0002	− 0.0002	− 0.0011
α_zz_	117.5032	143.0188	172.4628	74.1776	86.5840	99.6549
α_tot_	126.4701	147.2249	169.2656	123.8188	144.0192	165.4598
α_tot_ (in × 10^−23^ esu)	1.8742	2.1818	2.5085	1.8349	2.1343	2.4521
Hyperpolarisability (β_0_)	a.u			a.u		
β_xxx_	31.7753	79.4706	161.5415	− 268.1299	− 347.6121	− 425.2644
β_xxy_	− 2.6664	8.5946	31.9089	− 65.4170	− 110.3996	− 181.9322
β_xyy_	− 2.9491	11.1099	35.0638	83.3570	137.3193	222.7968
β_yyy_	− 4.4357	0.8323	32.6965	1.7223	− 22.1719	− 62.6067
β_xxz_	81.7811	100.8230	115.8360	− 0.0326	− 0.0553	− 0.0815
β_xyz_	− 5.6121	− 4.7809	− 11.2449	− 0.0067	− 0.0183	− 0.0387
β_yyz_	72.5230	93.2328	112.7677	− 0.0062	− 0.0201	− 0.0385
β_xzz_	− 35.6195	− 46.9268	− 64.8047	2.0848	6.3313	13.7875
β_yzz_	14.18962	15.8275	16.1746	24.3622	33.8174	46.6650
β_zzz_	176.2460	220.012305	252.1472	0.04223	0.0467	0.0494
β_tot_	330.6958	417.1280	504.9933	186.8742	226.6112	273.4122
β_tot_ (in × 10^−30^ esu)	2.8569	3.6036	4.3627	1.6144	1.9577	2.3620

The NLO parameters calculation was shown in Table 3; the dipole moment, mean polarizability (α_tot_,) and the first order hyperpolarizability (β_tot_) of nicotine and caffeine molecules are obtained in the basis set 6-311++G(d, p) with the CAM-B3LYP model. The dipole moments of both nicotine and caffeine are higher in DMSO solvent due to the highly polar nature of solvents. The dipole moments are also smaller in the CCl4 solvent than in the DMSO solvent and smaller still in the gas phase than in either of the solvents. The dipole moments of caffeine are higher than caffeine in all phases; this indicates caffeine is strongly polar. The polarizability comparison of nicotine is higher than that of caffeine in all phases. A higher α_tot_ value means that the electron cloud is more flexible, which means that the interactions between molecules are stronger. Also, the first order hyperpolarizability of the nicotine molecule is greater than that of the caffeine molecule in all phases. The higher value of β_tot_ shows the molecule is highly effective for NLO applications.

### Atoms in molecule (AIM)

The AIM analysis is used to determine the electronic structure and bonding of molecules. The bond critical points (BCPs), critical points (CPs), and interbasin paths of caffeine and nicotine molecules have been obtained by using the Multiwfn software 3.8 packages. The electron density and Laplacian of the electron density at the crucial locations are the most frequently employed standards for the presence of hydrogen bonding interactions^39^. The AIM provides valuable insights into specific molecule-system regions, including intermolecular or intramolecular interactions. Further employed to describe how strongly hydrogen bonds are formed.

**Fig. 5:**
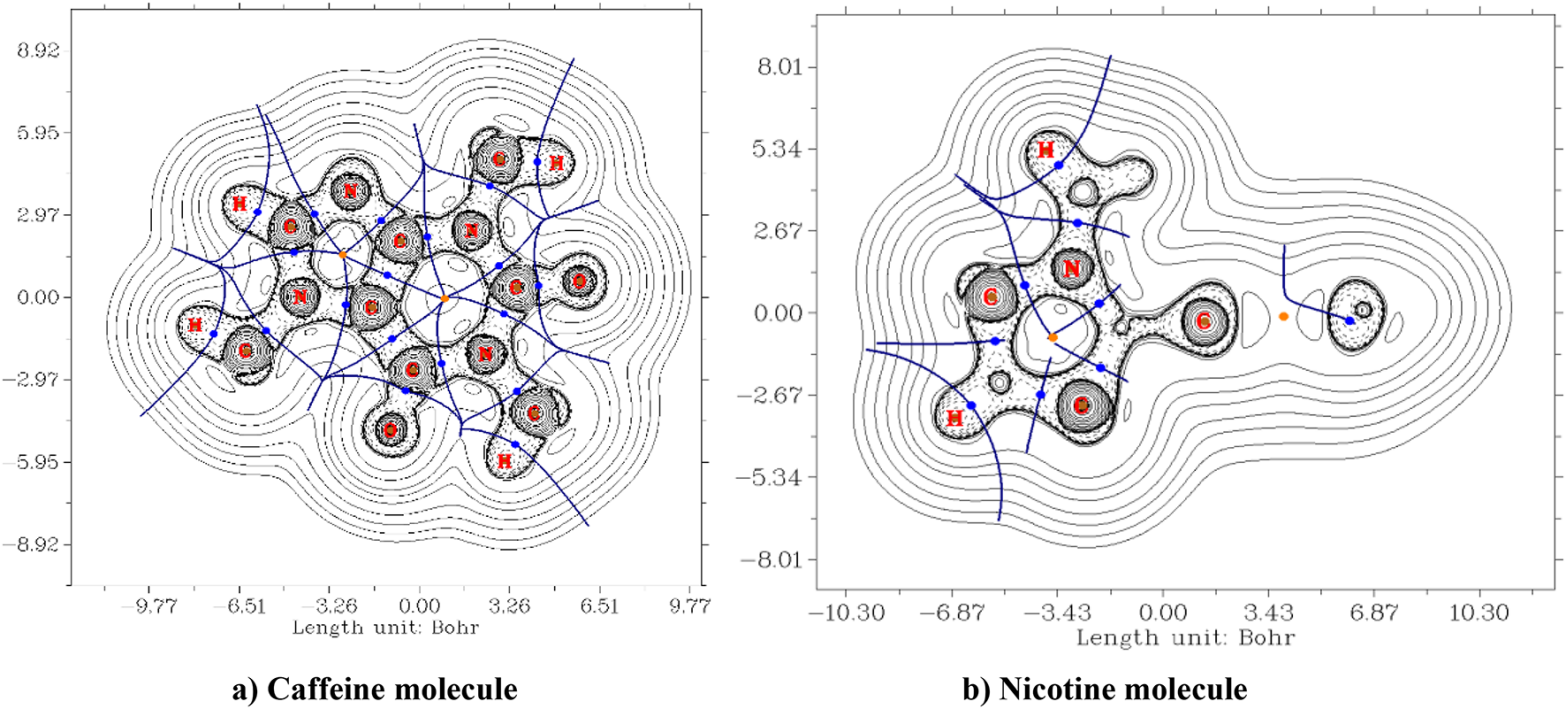
AIM of (**a**) caffeine in the XZ plane with a contour-filled map and (**b**) nicotine in the XY plane with a contour-filled map.

Bond critical points, or BCPs, determine the chemical bond and bond formation strength^40^. Brown, blue, and orange circles represent the critical points (CPs) in Fig. 5, while deep blue lines denote the interbasin path. Normally, topological parameters classify hydrogen bonds into strong, medium, and weak. Additionally, the contour plot of caffeine and nicotine molecules shows a solid line representing the positive region and a dashed line representing the negative region.

Figure 5a displays the contour map of electron density with topological paths of the caffeine molecule in the XY plane, while Fig. 5b displays the contour map of electron density with topological paths of the nicotine molecule in the XZ plane. The critical points, bond paths, and inter-basin paths are relatively more continuous than those of the nicotine molecules^41,42^. The caffeine molecule is more planar than the nicotine molecule.

### NCI-RDG analysis

**Fig. 6:**
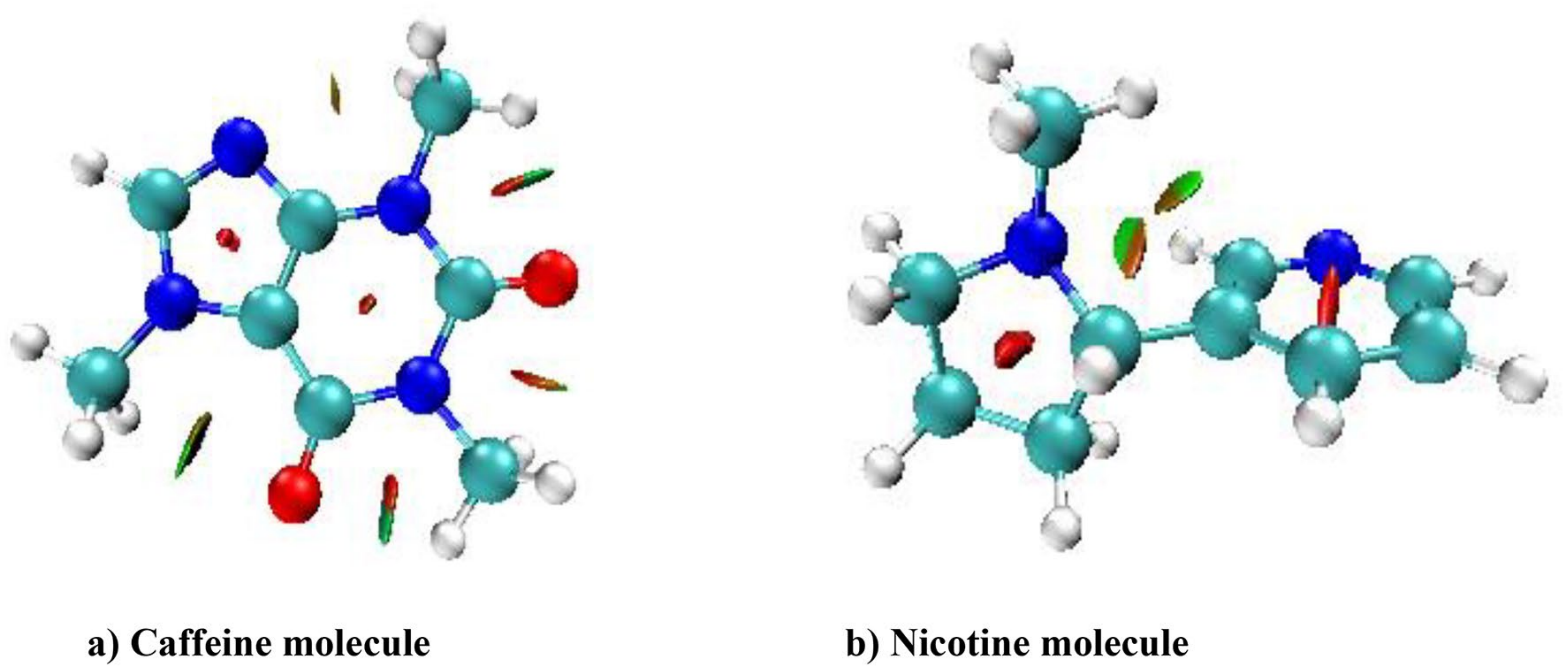
NCI of caffeine and nicotine molecule with H-bond (strong attraction), Van der Waals interaction, and steric effect (strong repulsion).

**Fig. 7:**
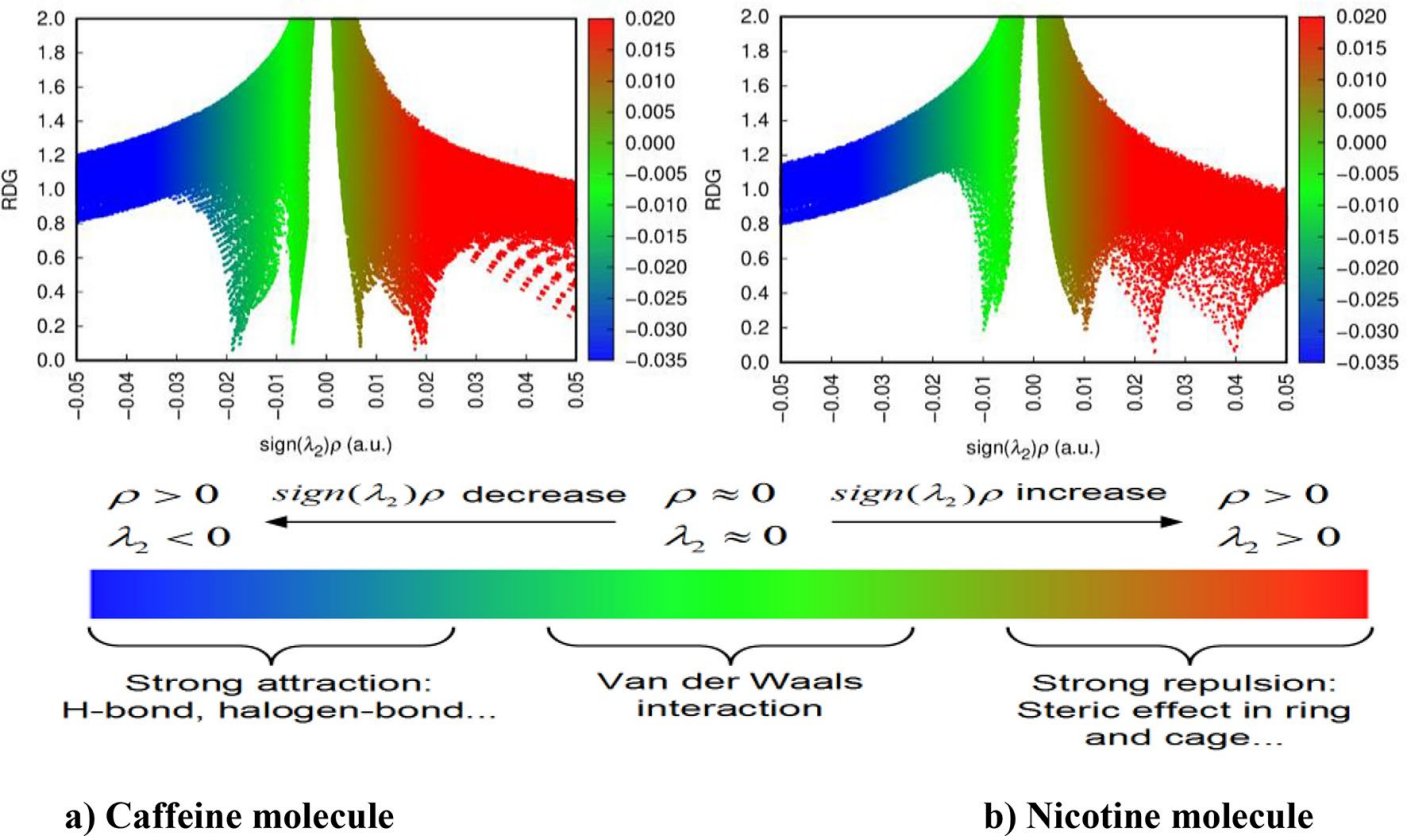
RDG scatter of (**a**) Caffeine and (**b**) Nicotine molecule with H-bond (strong attraction), Van der Waals interaction, and steric effect (strong repulsion).

Non-covalent interaction (NCI) enables the identification and visualization of non-covalent bonds. The molecular structure and reactivity of the molecule have been studied with the interaction of the molecule. RDG (reduced density gradient) is electron density and helps identify regions of non-covalent interaction. The NCI-RDG diagram is drawn by using Multiwfn 3.8 software^23^. Figures 6 and 7^43^ show the electron density, Van der Waals (VWD) interaction, steric effect, weak interaction in the molecular system, and hydrogen bond area of nicotine and caffeine molecules. The spikes in the RDG provide some information about the particular type of bond and the interaction’s strength^44,45^. The blue color (λ2 < 0) region denotes the H-bond (weak bond), the red color region (λ2 > 0) denotes the strongest repulsive interaction, and the green color (λ2 = 0) denotes the Van der Waals interaction.

### ELF, LOL and electron density

**Fig. 8:**
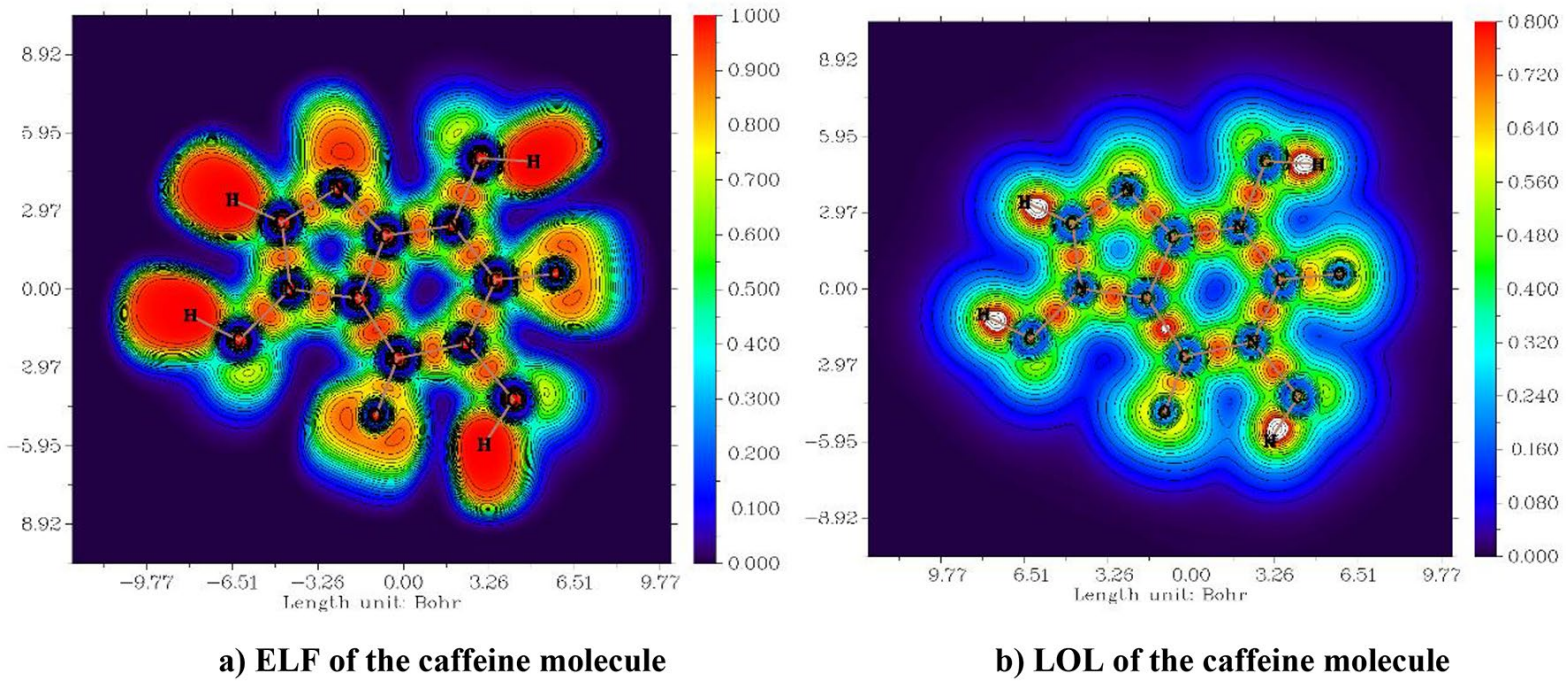
ELF and LOL in the XY plane of caffeine molecule with a contour-filled map.

**Fig. 9:**
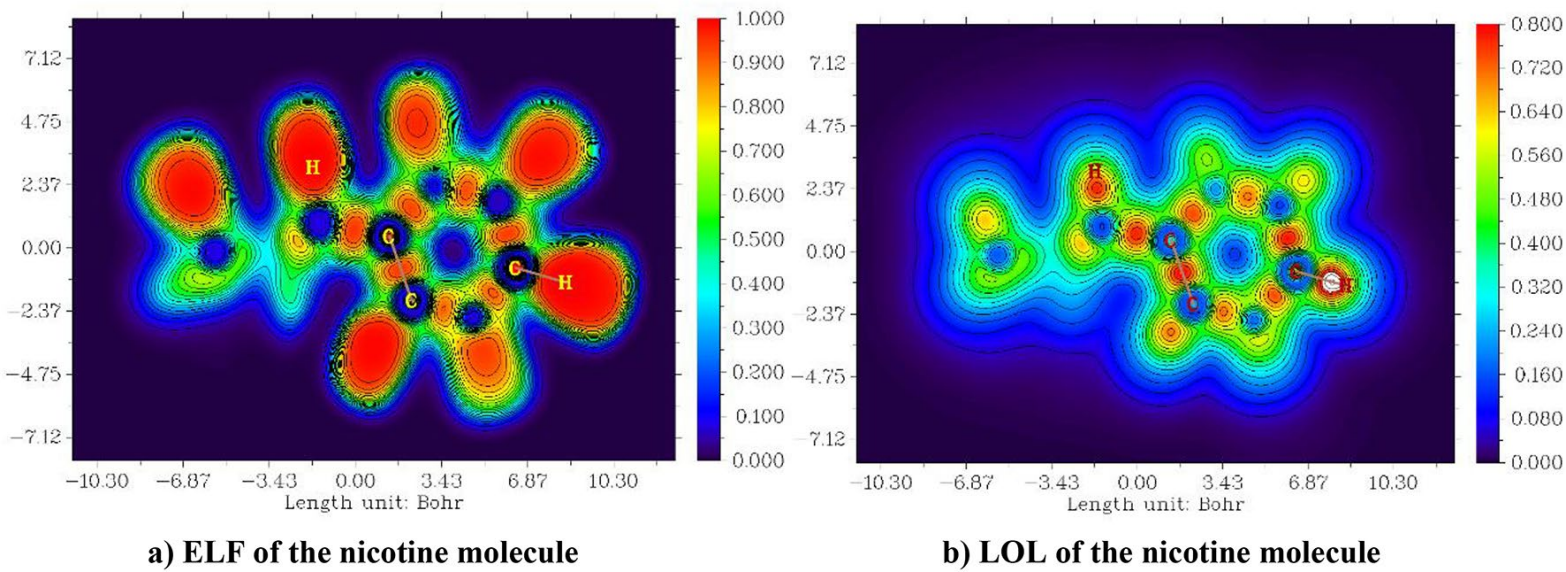
ELE and LOL in the XZ plane of nicotine with a contour-filled map.

Multiwfn software 3.8 draws the electron localization function (ELF) and localized orbital locator (LOL). The ELF and LOL demonstrate the atomic shell structure and classify chemical bonds and charge-shift bonds on the molecular surface through topological analyses^46^. LOL indicates that localized orbital gradients reach their maximum when localized orbitals coincide, while ELF relies on the concept of electron pair density, which considers the significant probability of finding an electron pair^47,48^. Figures 8 and 9 present the ELF and LOL of the caffeine and nicotine molecules, respectively. The surface analysis relies on the covalent bond between the ELF and LOL regions and the molecular surface^49^. The ELF is denoted by τ(r) and LOL is denoted by η(r). The ELF ranges from 0 to 1.0 in the XY plane, where 0.0 and 1.0 indicate the delocalization and localization of electrons^50,51^, respectively. Also (< 0.5) indicates delocalized. In the XZ plane, the LOL ranges from 0.0 to 0.8; a higher value (> 0.5) signifies the localization of high orbitals, corresponding to regions of bonding and lone pairs.

In caffeine, the molecule has plainer and almost localized four H atoms in ELF, but most of the C atoms are seen as delocalized atoms^52,53^. In LOL, we observe three H atoms and one N atom as localized atoms, while the remaining C atoms appear as delocalized atoms. Similarly, in both ELF and LOL, the nicotine molecule displays two localized H atoms and delocalized C atoms. The ELF and LOL of caffeine and nicotine molecules provide the atoms with both localized and delocalized regions. The ELF and LOL of these molecules also represent lone pairs and covalent bonds in localized regions (red), whereas the transformable electrons are represented by a delocalized blue color.

## Conclusions

The characterization of caffeine and nicotine molecules is carried out using the basis set 6-311++G(d, p) with the B3LYP and CAM-B3LYP methods in Gaussian 09 W software. The stability of caffeine and nicotine molecules with function B3LYP having minimum energy is − 18518.8 eV with dipole 3.98 Debye and − 13581.43 eV with dipole 2.97 Debye, respectively. Also, minimum energy has been calculated in different phases, like CCl4 and DMSO solvent. The solvent DMSO is more polar, and CCl4 is a non-polar solvent. The comparison of caffeine and nicotine shows that caffeine is more polar than nicotine due to their dipole moment. The interaction of the caffeine molecule with the protein chain exhibits polarity in nature. The geometrical optimization of these molecules with dipole moments using functions B3LYP and CAM-B3LYP has also been listed. The vibrational assignments of these molecules have been carried out, and Raman and UV-Vis spectra with ranges of wave numbers from 0 to 3500 cm^−1^ and 100–500 cm^−1^, respectively, have been obtained. The Raman spectra confirmed the different functional group presence in the molecular structure of caffeine and nicotine molecules. There are many more functional groups present in the nicotine molecule than in the caffeine molecule; the peak of the Raman spectra in caffeine is lower than the peak of the Raman spectra in the nicotine molecule. In UV-Vis spectra, the maximum peak in the caffeine molecule in CCl4 and DMSO solvent is found at 271.625 nm which is in good agreement with calculated data. Similarly, the maximum peak is found in the nicotine molecule at 258.994 nm in CCl4 solvent and 250.991 nm in DMSO solvent, but only agreement with the third excited state of calculated data. The comparison of NLO properties between caffeine and nicotine molecules in different phases, like gas phase, CCl4 and DMSO solvent. The nicotine molecule has higher polarizability and hyperpolarizability in all phases than the caffeine molecule. The topology and electron density were studied by using Multiwfn software 3.8; AIM has been carried out, which shows the critical points, bond critical points, and interbasin path of the caffeine and nicotine molecules. The NCI-RDG analyses were studied, which give the region of polar hydrogen and show the Van der Waals region of caffeine and nicotine molecules. Finally, we used ELF and LOL to calculate the electron density of caffeine and nicotine molecules. The red color region shows the maximum probability of electron confinements. These calculations are useful for designing the experiment and predicting the results from this calculation.

## Data Availability

The datasets used and/or analysed during the current study available from the corresponding author on reasonable request.

## Code availability

The relevant code with the manuscript is also available and would be available, if will be asked to do so later on reasonable request.
